# The Metropolitan Asylums Board and the Anti-Toxin Treatment

**Published:** 1895-02-09

**Authors:** 


### The Hospital

An Institutional Journal of

The Medical Sciences and Hospital Administration, with

Vol. XVII.—No. 437.] AN EXTRA NURSING SUPPLEMENT. [Feb. 9, 1895.

### THE METROPOLITAN ASYLUMS BOARD AND THE ANTI-TOXIN TREATMENT.

The medical journal which aspires to serve its generation with genuine loyalty must keep its eye steadily fixed on at least two objectives—the solid progress of medicine and the largest interests of the public. If we apply this rule to the recent proceedings and the immediate future purposes of the Metropolitan Asylums Board, it may help not only to illuminate the minds of the public, but also to guide the conduct of the Board.

The anti-toxin treatment of diphtheria, it will be admitted, involves the two questions of the public interest and the progress of medicine. That treatment, it is obvious even to the most rudimentary scientific sense, is still on its trial. The very latest reports we have on the subject, which come to us almost as we go to press, consist of the proceedings of the Vienna Medical Society. At the last meeting of that society Professor Drasche announced certain conclusions he had arrived at after personal observation of thirty cases of diphtheria treated by serum anti-toxin. He affirmed two things: First, that the anti-toxin serum had affected the kidneys in certain cases; and, secondly, that, notwithstanding the use of the serum, the total mortality from diphtheria in Berlin and Vienna had not been diminished. Drasche's observation, that in his cases the kidneys had been affected, was corroborated by those of other physicians present. Summarising his conclusions still further, the learned professor said that in all the thirty cases the progress of which he had followed he had not met a single instance in which he could feel convinced that the treatment had produced a direct effect. The disease developed in an exactly similar manner in those patients who were treated with the serum as in those who were not. On the other hand, there were many symptoms which warned medical men against its use.

That, of course, is what criticism, carried to its utmost legitimate limits, has to say. Professors Kolisko and Paltauf, with Dr. Gruber and others, firmly defended the new remedy. They had no evidence of a fresh character to bring to bear upon the controversy. Their reliance was placed upon statistics. It is manifest that we need not repeat their contentions verbatim, since, if statistics alone are to be trusted to decide the matter, it is decided already, and that altogether in favour of anti-toxin.

Two questions may here be asked: Can the Metropolitan Asylums Board render an important service to medicine and the public at this conjuncture? And, if it can, has the public any right to demand that it shall? Both these questions, it seems to us, must be answered in the affirmative. The Clinical Society is apparently of the same opinion. It has appointed a committee to investigate and report upon the value of anti-toxin in the treatment of diphtheria, and it has addressed a letter to the Metropolitan Asylums Board requesting permission to apply to the medical superintendents of the Board's hospitals for particulars of their observation of the use of the alleged remedy in those institutions. One of the members of the Board proposed that the permission asked should be granted forthwith; but Dr. Stamford Felce, with a juster sense of responsibility, moved an amendment, which was ultimately adopted, to the effect that the letter should be referred to the General Purposes Committee for consideration and report. There, we suppose, the matter now stands.

It is probable that many persons, as well as ourselves, who are vigilantly following the modern developments of practical medicine, have made up their minds as to the course which is most desirable at this conjuncture. Co-operation is the note which should be firmly struck. The Clinical Society should not only be granted the permission it has asked, but it should be invited by the authorities of the Metropolitan Asylums Board to take an active part in the investigation in which the medical office[illegible] of the Board are engaged. But in order to make the proposed research worthy of public confidence in the highest possible degree, the committee of investigation should be made still more large and representative. The Patholo[illegible]ical as well as the Clinical Society should be asked to appoint delegates, as also should the Colleges of Physicians and Surgeons, and the British Medical Association. A representative committee thus constituted would command the entire confidence of both the medical professio nand the public in this country, and would, we venture to think, within a reasonable period of time, arrive at conclusions on this important public question which would be generally accepted throughout the civilised world.

The Metropolitan Asylums Board has a conspicuous opportunity before it, and we can hardly imagine that it will decline to accept the honourable responsibility. No doubt there may be difficulties and routine obstructions, but that is a mere matter of course. The question is, which will serve the public interest best, a full investigation such as that proposed by the Clinical Society, or the avoidance of such an investigation in deference to routine and obstruction of a mere *laissez faire* character? There can, we think, be but one answer to this question. It is eminently desirable, in the interests alike of medical progress and the public well-being, that the Metropolitan Asylums Board shall find the right answer, and that with promptitude.

